# Energy and Dynamics of Caveolae Trafficking

**DOI:** 10.3389/fcell.2020.614472

**Published:** 2021-01-21

**Authors:** Claudia Matthaeus, Justin W. Taraska

**Affiliations:** Biochemistry and Biophysics Center, National Heart, Lung and Blood Institute, National Institutes of Health, Bethesda, MD, United States

**Keywords:** caveolae, caveolin, dynamin, EHD2, membrane trafficking, lipid trafficking

## Abstract

Caveolae are 70–100 nm diameter plasma membrane invaginations found in abundance in adipocytes, endothelial cells, myocytes, and fibroblasts. Their bulb-shaped membrane domain is characterized and formed by specific lipid binding proteins including Caveolins, Cavins, Pacsin2, and EHD2. Likewise, an enrichment of cholesterol and other lipids makes caveolae a distinct membrane environment that supports proteins involved in cell-type specific signaling pathways. Their ability to detach from the plasma membrane and move through the cytosol has been shown to be important for lipid trafficking and metabolism. Here, we review recent concepts in caveolae trafficking and dynamics. Second, we discuss how ATP and GTP-regulated proteins including dynamin and EHD2 control caveolae behavior. Throughout, we summarize the potential physiological and cell biological roles of caveolae internalization and trafficking and highlight open questions in the field and future directions for study.

## Introduction

**Figure 1:**
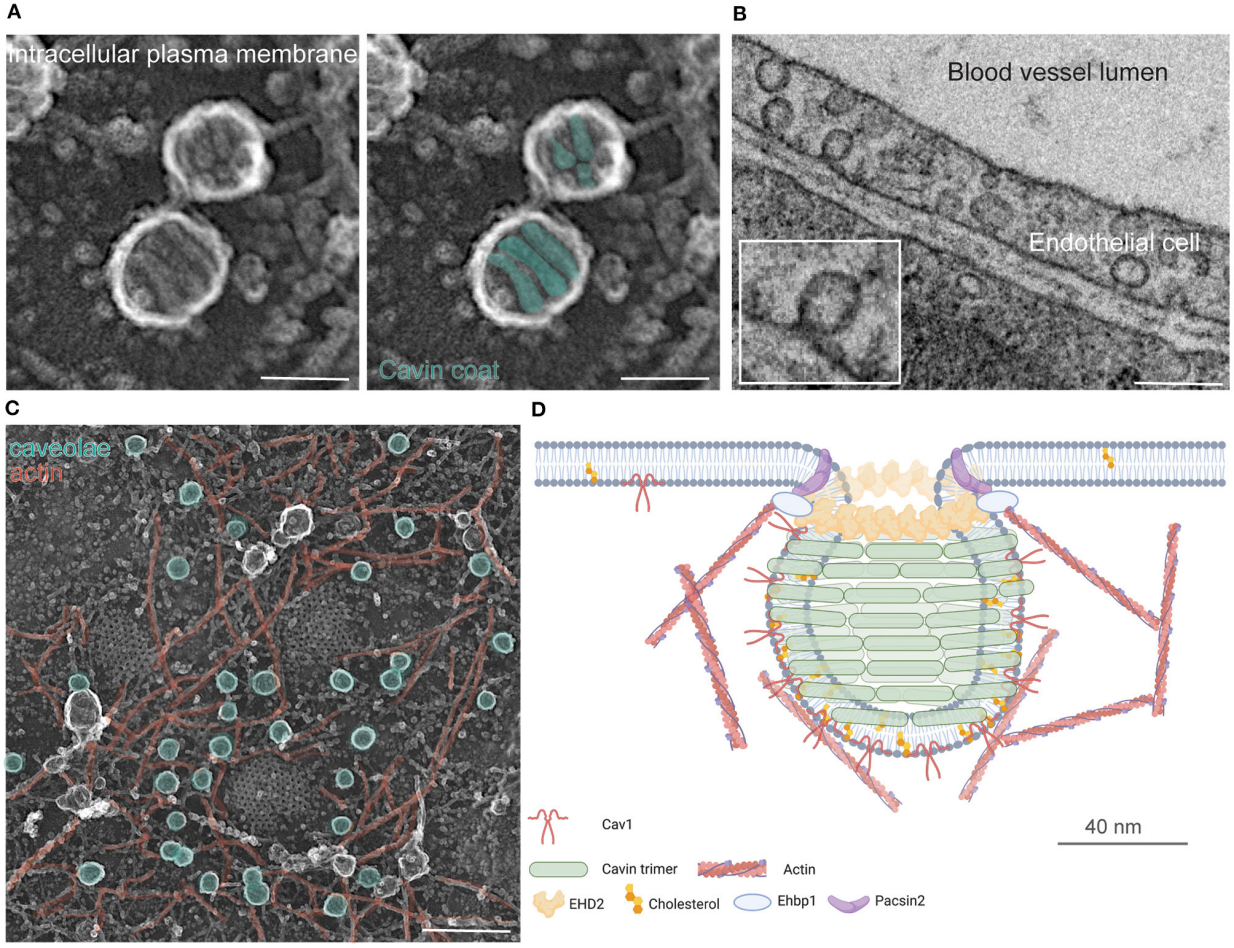
Caveolae structure and components. **(A)** Caveolae imaged in unroofed mouse embryonic fibroblasts (MEF) by platinum replica EM. The typically caveolae coat generated by Cavin protein complexes (colored in green) surround the Cav1 induced plasma membrane invaginations. Scale bar 120 nm. **(B)** 2D TEM image of caveolae in endothelial cells of blood vessels in mouse heart sections. Scale bar 200 nm. **(C)** The MEF plasma membrane contains randomly distributed caveolae (in green) embedded with actin filaments (red). Scale bar 250 nm. **(D)** Schematic overview of caveolae and its components.

Caveolae are 70–100 nm diameter sized plasma membrane invaginations that form bulb shape invaginations into the cytosol (Figure 1). They are found in a variety of cell types including adipocytes, endothelial cells, muscle cells, fibroblasts, and astrocytes (Cameron et al., 1997; Parton, 2003; Parton and Del Pozo, 2013; Parton et al., 2018; Yan et al., 2019). The plasma membranes of endothelial, muscle, and fat cells are packed with caveolae, suggesting an important role in specialized functions including homeostasis and metabolism. Caveolae also comprise a specific lipid environment containing large amounts of cholesterol, sphingomyelin, and ceramides (Graf et al., 1999; Parton et al., 2020b; Zhou et al., 2020). These lipids accumulate in caveolae, providing a reservoir for these molecules (Hubert et al., 2020b). Therefore, these organelles serve as unique scaffolds for plasma membrane proteins involved in signaling pathways creating unique cell-type specific protein signaling domains. Caveolae are also known to participate in cellular lipid and fatty acid uptake (Pilch and Liu, 2011; Pilch et al., 2011), endothelial transcytosis of large molecules (Frank et al., 2009; Cheng and Nichols, 2016), regulation of the endothelial NO synthase (Förstermann and Sessa, 2012), neurovascular coupling (Chow et al., 2020), viral internalization (Pelkmans et al., 2001; Xing et al., 2020), and pigmentation in melanocytes (Domingues et al., 2020). Furthermore, under some cellular membrane tension regimes caveolae can group into larger clusters at the plasma membrane termed caveolae rosettes (Echarri et al., 2019; Golani et al., 2019). The wide array of structures and actions of caveolae across many different tissues and pathways highlights this small organelle's diverse role in signaling and physiology. Yet, much remains to be uncovered about their regulation, function, and mode of action.

The caveolar coat is minimally composed of several proteins important for forming and stabilizing the bulb-shaped membrane invagination. The key proteins are Caveolin (three orthologous in human, Cav1-3), Cavins (Cavin1-4), the BAR protein domain-containing syndapin/Pacsin2, and the dynamin-related ATPase EHD2 (see Figure 1, previously reviewed in the literature; Han et al., 2016b; Lamaze et al., 2017; Parton et al., 2020c). *In vitro* and *in vivo* studies have shown that Caveolin/cholesterol complexes incorporate into the plasma membrane forming elongated and rather shallow invaginations (Fernandez et al., 2002; Hayer et al., 2010a; Ariotti et al., 2015; Han et al., 2020). Caveolin complexes alone do not form the typical bulb shape. Therefore, Cavin coat proteins have been proposed to be essential for generating the classic “cave-like” invaginations. Specifically, Cavins are recruited from the cytosol, oligomerize into trimers, and surround the caveolar membrane resulting in a structured caveolar coat as illustrated in Figure 1A (Gambin et al., 2014; Kovtun et al., 2015; Ludwig et al., 2016; Stoeber et al., 2016). Furthermore, it has been shown that Pacsin2 is important for bending the membrane and stabilizing caveolar invaginations at the plasma membrane (Hansen et al., 2011; Senju et al., 2011, 2015; Seemann et al., 2017). A similar function was found for the ATPase EHD2. EHD2 specifically localizes to the neck of caveolae (Morén et al., 2012; Stoeber et al., 2012; Ludwig et al., 2013). Recently, the EHD2 binding partner (EHBP1) was identified as another stabilizer of caveolae in endothelial cells (Webb et al., 2020). Finally, a BAR protein, FBP17, was discovered to be important for the formation of caveolae rosettes at the plasma membrane (Echarri et al., 2019).

Despite the large number of caveolae in muscle and endothelial cells or adipocytes, the loss of caveolae due to Cav1 or Cavin1 deletion is not generally lethal (overview of knockout models reviewed in Le Lay and Kurzchalia, 2005; Hansen et al., 2013; Cheng and Nichols, 2016). However, investigations of various Caveolin or Cavin deficient mouse models has indicated impaired lipid metabolism and lipodystrophy, cardiomyopathies, blood pressure changes, and muscular dystrophy in these animals (Pilch and Liu, 2011; Cheng and Nichols, 2016). It should be noted that some of these phenotypes could be due to non-caveolar functions of Cav1 as recently reviewed (Pol et al., 2020). Additionally, altered Cav1 and Cavin1 expression, mutations in human Caveolin genes, as well as modified caveolae endocytosis and trafficking can be linked to metabolic diseases including obesity and lipodystrophy (Catalán et al., 2008; Kim et al., 2008; Pilch and Liu, 2011; Schrauwen et al., 2015; Matthaeus et al., 2020), cancer (Lee et al., 2002; Martinez-Outschoorn et al., 2015; Ketteler and Klein, 2018) as well as cardiovascular diseases (Cohen et al., 2004a; Han et al., 2016a; Lian et al., 2019) or myopathies (Gazzerro et al., 2010; Dewulf et al., 2019). Therefore, caveolae are currently under investigation as novel therapeutic targets for disease (Carver and Schnitzer, 2003; Navarro et al., 2014).

The process of intracellular membrane traffic, including caveolae endocytosis, transcytosis, transport, and targeting, requires specific signals and regulatory modules to direct movement inside the cell. Nucleoside triphosphates Adenosine-5'-triphosphate (ATP) and Guanosine-5'-triphosphate (GTP) serve as cellular energy resources to drive, localize, and direct these actions. Both nucleosides play essential roles during intracellular trafficking to promote membrane interactions and deformations, protein-protein interactions, and conformational changes in molecular machines. Here, we summarize the state of understanding of caveolae membrane trafficking and highlight the roles of ATP and GTP-dependent processes within these specialized membrane structures.

## Caveolae Trafficking

**Figure 2:**
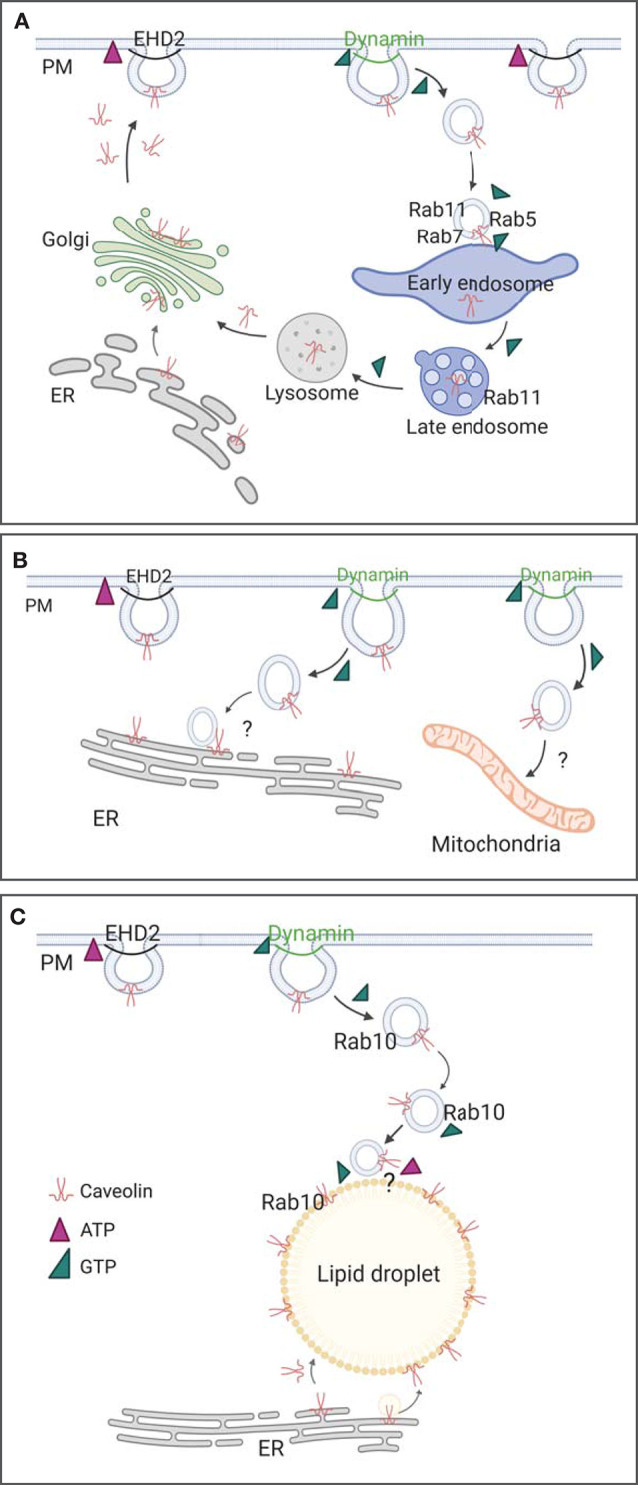
Caveolae endocytosis and trafficking. **(A)** Classic caveolae endocytosis from the plasma membrane to early and late endosomes, followed to lysosomes, occurs in various cell types. Rab5, 7, and 11 are assigned to caveolae trafficking, ATP (pink triangle) and GTP (green triangle) dependent processes are illustrated. **(B)** Caveolae trafficking to endoplasmic reticulum (ER) and mitochondria is suggested based on initial proteomics data and detailed EM images. **(C)** Caveolae trafficking from the plasma membrane to lipid droplets can be observed in adipocytes and fibroblasts. PM, plasma membrane; Dyn, Dynamin; ER, endoplasmic reticulum; question mark indicates unknown processes and proteins.

How do caveolae move? Caveolae endocytosis and trafficking has been observed in many cell types although the cellular consequences of these movements are currently not well-understood. The role and even occurrence of caveolae traffic has, indeed, been controversial (Parton and Howes, 2010; Cheng and Nichols, 2016; Parton et al., 2020a). Several studies have shown, however, that caveolae endocytosis supports viral entry and receptor internalization, and caveolae membrane trafficking has been linked to cellular lipid homeostasis and movement. Here, we divide caveolar internalization into 5 steps: (1) caveolae dynamics at the plasma membrane, (2) detachment from the cell membrane, (3) fusion with endosomes followed by accumulation in lysosomes or (4) non-endosomal trafficking to intracellular organelles, and finally (5) recycling of caveolae (see overview in Figure 2 and the following sections).

With these steps in mind, caveolae internalization and traffic, however, must be distinguished from caveolae flattening and disassembly. Several studies have indicated that increased membrane tension due to osmotic shock and membrane stretch could lead to caveolar membrane flattening. Here, caveolae proteins such as Cavins or EHD2 are proposed to be released into the cytosol and are able to move independently of caveolar membranes (Sinha et al., 2011; Cheng et al., 2015; Garcia et al., 2017; Lim et al., 2017; Torrino et al., 2018). However, this mechano-adaptive caveolae behavior is cargo-independent and therefore different from classical endocytosis and traffic (Del Pozo et al., 2021). Recently, it was shown that cellular stress induced by UV light could also trigger the disassembly of caveolae and the release of caveolar proteins into the cytosol (McMahon et al., 2019). Previous studies further showed that caveolae serve as cellular membrane tension sensors that are coupled to mechano-transduction pathways such as the Hippo system involving the transcriptional regulators YAP and TAZ (Echarri and Del Pozo, 2015; Dewulf et al., 2019; Del Pozo et al., 2021). Here, caveolae are able to sense changes in plasma membrane tension and induce transcriptional changes as well as provide membrane reservoirs to protect the cell from mechanical stress. These behaviors of caveolae are distinct from the classic traffic routes we will focus on in this review.

The study of caveolae trafficking has been complicated by many technical difficulties. To investigate caveolae dependent endocytosis, cholera toxin and simian virus 40 have been the standard cargo. Yet, detailed analysis has shown that both cargos are not exclusively internalized by caveolae. This has led to some amount of conflicting data (Nichols, 2002; Cheng and Nichols, 2016). To date, no caveolae-specific cargo has been identified (Parton et al., 2020a). This makes it challenging to specifically monitor intracellular caveolae trafficking. Furthermore, non-caveolar Cav and Cavin localization, and overexpression effects of Cav/Cavin proteins, makes it difficult to distinguish between intracellular Cav/Cavin protein migration and caveolae endocytosis (summarized for Cav1 in Pol et al., 2020, non-caveolar Cavin function see Jansa et al., 1998; Liu and Pilch, 2016; McMahon et al., 2019). Additionally, the comparison of Cav1 overexpressing cells with genome-edited Cav1 cells revealed that only a portion of caveolae are actually motile and the majority stay immobile at the plasma membrane (Shvets et al., 2015). In contrast, overexpressed GFP-tagged Cav1 results in many highly mobile Cav1 molecules in cells which may not be assigned to caveolae migration. Therefore, caveolae endocytosis studies should be evaluated with care. Specifically, untangling the movement of coated caveolae membrane-containing vesicles from packets of caveolae proteins without membrane is a challenge. The following section will summarize current caveolae trafficking concepts while highlighting the distinct steps of the process.

### Caveolae Detachment From the Plasma Membrane

Caveolae are highly dynamic membrane domains, that are capable of moving laterally in the plasma membrane, similar to lipid rafts (Pelkmans and Zerial, 2005; Boucrot et al., 2011; Shvets et al., 2015). However, it was shown *in vitro* and *in vivo* that EHD2 localizes to caveolar necks. In particular, the observation of an increased number of static caveolae in cells overexpressing EHD2 led to the hypothesis that EHD2 stabilizes caveolae at the plasma membrane (Morén et al., 2012; Stoeber et al., 2012; Ludwig et al., 2013; Matthaeus et al., 2020). Besides EHD2, Pacsin2 is also involved in caveolae plasma membrane stabilization (Senju et al., 2011, 2015). Deletion of either EHD2 or Pacsin2 results in increased caveolae mobility and internalization. Most likely, EHD2 removal from caveolar neck promotes caveolae detachment from the plasma membrane followed by intracellular trafficking. How exactly detachment occurs is currently unknown. Dynamin has been proposed to be important for this process (Henley et al., 1998; Oh et al., 1998, 2012). Yet, clear evidence that dynamin acts alone or in combination with other proteins, such as intersectin (Predescu et al., 2003, 2012), is lacking. It is also possible that the fission of caveolae from the plasma membrane is driven by other proteins. Indeed, this is common for dynamin independent endocytosis (e.g., CLICs, Mayor et al., 2014; Sathe et al., 2018) and ESCRT III complex protein driven invaginations (Hanson et al., 2008; Rossman and Lamb, 2013).

To study caveolae scission and trafficking, one needs to differentiate vesicles from membrane bound caveolae. In the past this has been difficult for various endocytic pathways due to their small size and fast dynamics. In fixed cells, however, caveolar vesicles can be distinguished by EM or electron tomography as vesicles containing an enclosed lipid bilayer (Popescu et al., 2006; Hubert et al., 2020b; Matthaeus et al., 2020; Webb et al., 2020). Novel super resolution imaging techniques such as Stochastic Optical Reconstruction microscopy (STORM) and Stimulated Emission Depletion (STED) microscopy with resolution limits up to 40 nm are also able to identify caveolar vesicles (Platonova et al., 2015; Tachikawa et al., 2017; Yeow et al., 2017; Khater et al., 2018; Matthaeus et al., 2019). Of special interest with regard to caveolar vesicle trafficking is the application of STED and structured-illumination microscopy (SIM) to live cells allowing single caveolae to be tracked throughout the cell. Currently, caveolae trafficking is mainly studied with total internal reflection fluorescence (TIRF) microscopy. However, TIRF is diffraction limited in the plane of the cover glass and in the axial plane limited to signals within ~200 nm of the plasma membrane. Therefore, deeper caveolae events cannot be seen. In summary, rapidly developing imaging techniques will help to further elucidate the exact caveolar detachment process at the plasma membrane and allow single organelles to be monitored as they move through the cytosol. These studies will finally reveal how caveolae move, where they go, and what pathways they participate in within living cells and tissues.

### Intracellular Caveolae Trafficking

After detachment from the plasma membrane, caveolae can be internalized and traffic to intracellular organelles. Which signaling events, cargos, or ligands induce caveolae internalization are, however, unclear and has led to some controversy in the field. Thus, the exact role for caveolae traffic has been difficult to generalize. This is in contrast to clathrin mediated endocytosis where defined cargos and trafficking routes are well-established and mainly accepted. Recent data has suggested that high levels of extracellular cholesterol and glycosphingolipids are able to stimulate caveolar dynamics (Hubert et al., 2020b). Also, albumin (Minshall et al., 2000; Botos et al., 2008), okadaic acid and glycosphingolipids (Parton et al., 1994; Shvets et al., 2015), cholesterol and long-chain fatty acids (Le Lay et al., 2006; Hao et al., 2020), simian virus 40 (Tagawa et al., 2005), and endothelin (Oh et al., 2012) are thought to be potential detachment and internalization triggers in some cell types. Taken together, various tissue and cell specific signaling events or ligands may trigger caveolae internalization. The resulting intracellular caveolae trafficking routes include the conventional endocytic pathway, as well as caveolae migration to the endoplasmic reticulum and lipid droplets (see overview in Figure 2, and detailed description below). Likewise, studies have shown that caveolae detachment and return of caveolae vesicles to the plasma membrane can occur (Pelkmans and Zerial, 2005; Hubert et al., 2020a).

#### Endocytic Caveolae Pathway

When caveolae bud off from the plasma membrane they can fuse with the early endosome. This is followed by maturation of these organelles into late endosomes, multivesicular bodies, and finally degradation of their contents within lysosomes (Hayer et al., 2010b; Shvets et al., 2015). Previous studies showed that Cav1 co-localizes with early and late endosomal markers including Rab5 and Rab7, followed by accumulation into the lysosomes (Pelkmans et al., 2004; Botos et al., 2008; Hayer et al., 2010b; Shvets et al., 2015). Notably, Shvets et al. (2015) determined that caveolae mediated endocytosis in 3T3 fibroblasts comprises a minor fraction of total cellular endocytosis (ca. 5–10% of total endocytic vesicles). By using Cav1 immunogold labeling, Botos et al. (2008) observed an accumulation of Cav1 in multivesicular bodies after detachment from the plasma membrane. Furthermore, polarized epithelial cells contain Cav1 endocytosis and co-localization with the specific apical recycling marker Rab11a (Lapierre et al., 2012). In line with these observations, several proteomics and biochemistry studies showed an enrichment of small Rab GTPases, SNAP molecules, and the vesicle SNARE protein VAMP2 in isolated caveolae fractions (Schnitzer et al., 1995; Aboulaich et al., 2004; McMahon et al., 2006; Wypijewski et al., 2015). These are necessary for a functional membrane fusion machinery needed for the endocytic pathway. In summary, these data illustrate that caveolae are endocytosed. Most likely, viruses such as the simian virus 40 use this caveolar endocytic path way to enter and infect cells (Pelkmans et al., 2001; Tagawa et al., 2005). Furthermore, receptor internalization may be regulated by this pathway, e.g., TGF-beta type 1 (He et al., 2015) or insulin receptor (Fagerholm et al., 2009), although distinct caveolar specific receptors have not been observed.

#### Caveolae Trafficking to Endoplasmic Reticulum and Mitochondria

Recent advances in imaging and proteomics have uncovered novel caveolae trafficking routes outside the classic endocytic pathway. First, based on the observation of caveolae dependent cholera toxin and autocrine motility factor accumulation in the endoplasmic reticulum (ER) and Golgi, it was suggested that caveolae are able to migrate from the cell surface to the ER (Pelkmans et al., 2001, 2004; Le and Nabi, 2003). Additionally, proteomics of isolated caveolar membrane domains showed an increased amount of ER related proteins (McMahon et al., 2006). Recently, it was shown that Cav1 impairs the formation of ER-mitochondria contact sites and is involved in Drp1 mediated mitochondria fusion (Bravo-Sagua et al., 2019). However, clear evidence of Cav1 and/or caveolae originating from the plasma membrane under these circumstances is lacking. Indeed, the work of Bravo-Sagua et al. (2019) indicates a specific ER related function of Cav1 independent of caveolae endocytosis.

Caveolae have been proposed to form specific ER membrane contact sites. A detailed high resolution EM analysis of rat smooth muscle cells indicated that the majority of caveolae (either located at the plasma membrane or detached) are close to the sarcoplasmic reticulum (SR) (Popescu et al., 2006). The authors further detected electron densities in the caveolae membrane reaching to the corresponding SR membrane and containing potential tethers that establish membrane contact sites. The same observation was found for mitochondria, although caveolae-mitochondria contact sites are less abundant (Popescu et al., 2006). Currently it is not known if caveolae migrate from the plasma membrane to mitochondria. However, previous studies showed that Cav1 is also found in mitochondria (Li et al., 2001; Fridolfsson et al., 2012; Foster et al., 2020).

#### Caveolae Trafficking to Lipid Droplets

Caveolae trafficking to lipid droplets is of particular interest for lipid homeostasis and metabolism. Initially, Cav1 and independently Cav2, were found at lipid droplets. It was suggested that Cav1 or Cav2 originated from the ER and migrates to lipid droplets because pharmacological inhibition of ER vesicle transport was seen to block Cav translocation (Fujimoto et al., 2001; Ostermeyer et al., 2001; Pol et al., 2001, 2004). By overexpressing a Cav1 mutant leading to ER accumulation, Cav1 re-locates to the lipid droplet coat, most likely during lipid droplet formation (Ostermeyer et al., 2004; Pol et al., 2004). The lipid droplet coat consists, in contrast to other membrane-bound organelles, of a phospholipid monolayer, resulting in a unique set of proteins targeted to this area (Walther and Farese, 2009; Kory and Walther, 2016). The recruitment and localization of proteins directly from the cytosol to the lipid droplet coat requires amphipathic helices to ensure correct localization (Kory and Walther, 2016). Caveolins contain amphipathic lipid binding domains and therefore are likely able to bind to lipid droplets (Ariotti et al., 2015; Root et al., 2019).

Cav1 trafficking from the plasma membrane to lipid droplets was first described in 3T3-L1 adipocytes using immunostaining, EM, and biochemistry (Le Lay et al., 2006; Blouin et al., 2010). Blouin et al. (2010) further detected Cavin1, EHD2 and semi-carbazide-sensitive amine oxidase (SSAO, localizes within the adipocyte caveolae domain, Souto et al., 2003) at the lipid droplet coat indicating that not Cav1 alone but caveolae are recruited to lipid droplets. Interestingly, Cav1 (most likely Cav2 as well) is recruited to a specific lipid droplet subpopulation (Storey et al., 2011). The isolation and purification of lipid droplets revealed a Cav1 positive lipid droplet fraction that is also enriched in Perilipin1, a protein protecting stored lipids within the droplets against lipolysis (Sztalryd and Brasaemle, 2017). In contrast, Cav1 negative droplets showed an accumulation of ADRP (adipocyte differentiation-related protein, Storey et al., 2011). Furthermore, Cav1 and Perilipin1 are able to form a complex, indicating that Cav1 is involved in the regulation of lipolysis of the lipids stored in the lipid droplets (Cohen et al., 2004b; Storey et al., 2011).

These data suggest that there is direct trafficking from the plasma membrane. However, how this is carried out and if Cav1 alone or caveolae vesicles are transferred is currently not understood in mechanistic detail. Recent data from Matthaeus et al. (2020) further supported this idea as increased caveolae mobility and endocytosis resulted in increased lipid droplet size and increased fatty acid uptake. Additionally, lipid accumulation within caveolar membrane domains increased caveolae detachment (Shvets et al., 2015; Hubert et al., 2020b) indicating the importance of this process. Based on these data, we propose the following model. Caveolae may serve as lipid sensors that accumulate specific lipids such as cholesterol, sphingolipids, and fatty acids. By reaching a critical amount of lipids, the stability of membrane-attached caveolae decreases and caveolae detach. This sensing is followed by caveolae traffic to lipid droplets. However, it is currently unclear how caveolae sense the accumulation of lipids within their membrane domains, how EHD2 disassembles, and how this scission is regulated. It is also unclear if caveolae migrate as vesicles to the lipid droplet coat and form membrane contact sites (such as shown for other organelles, reviewed by Olzmann and Carvalho, 2019; Thiam and Dugail, 2019; Henne, 2020), or if Cav1 alone is able to reach them.

Taken together, there is much data illustrating caveolae internalization from the plasma membrane followed by intracellular trafficking. Currently, the conventional pathway is the best studied. Yet, proteomic approaches suggest caveolae target to other organelles and these must be evaluated in detail. Super resolution imaging techniques can help to track the global movements of caveolae throughout cells. Besides the different intracellular caveolae trafficking pathways, the initial steps, ATP dependent EHD2 stabilization at the plasma membrane, and the GTP dependent dynamin-based scission of the caveolar bulbs, are all essential for caveolae internalization. Next, we discuss the function and importance of the energy-dependent enzymes including EHD2 and dynamin in these specific processes.

## ATP Dependent EHD2 Oligomerization at the Caveolar Neck

How is EHD2 oligomerization and its effect on caveolae plasma membrane stabilization and detachment regulated? Recent structural, cellular, and physiological data indicated that the ATP-dependent oligomerization of EHD2 is an important regulator for caveolae traffic. Eps15 homologous domain containing protein 2 (EHD2) and its related EHD proteins belong to the dynamin protein family as they share the same overall domain organization (Daumke et al., 2007). In mammals, four EHD orthologs are found (EHD1-4) that show distinct tissue-specific expression, localization, and functions (Pohl et al., 2000; George et al., 2007). EHD1, 3 and 4 are observed at early and late endosomes and EHD2 is located primarily at the caveolar neck (reviewed in Naslavsky and Caplan, 2011; Bhattacharyya and Pucadyil, 2020). All EHD proteins are able to bind to and bend phospholipid membranes (Daumke et al., 2007; Melo et al., 2017; Deo et al., 2018).

**Figure 3:**
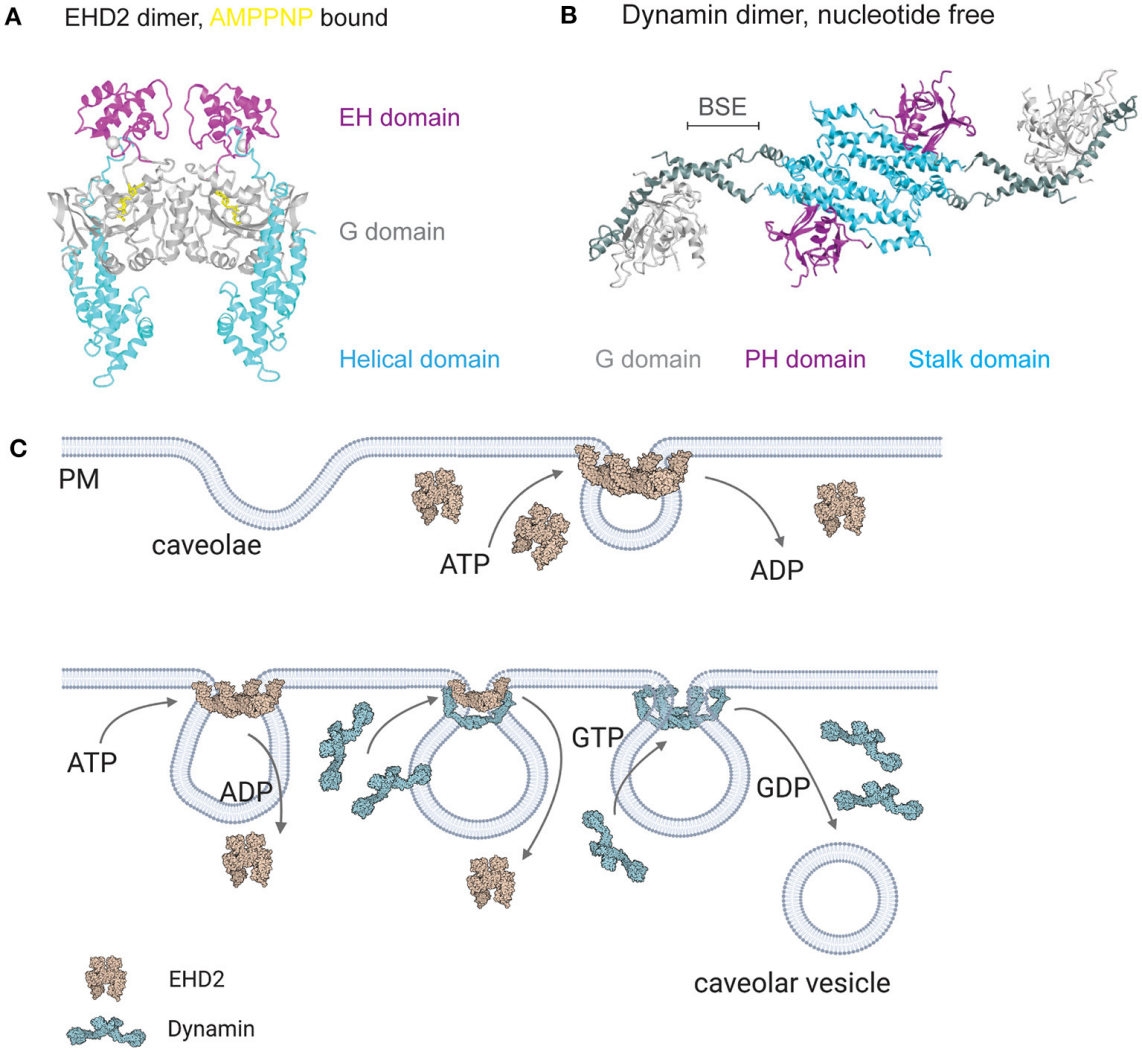
EHD2 and dynamin at the caveolar neck. **(A)** Crystal structure of EHD2 dimer bound with non-hydrolyzing AMPPNP (PDB 2QPT, Daumke et al., 2007). **(B)** Crystal structure of nucleotide free dynamin dimer (PDB 3SNH, Faelber et al., 2011). **(C)** ATP and GTP dependent caveolae stabilization and detachment. After caveolae formation, EHD2 oligomerizes in an ATP dependent manner in large rings at the neck of caveolae. Disassembly of EHD2 oligomers occurs after ATP hydrolyzation, followed by dynamin recruitment to the caveolar neck and GTP dependent membrane scission. Membrane binding of EHD2 and dynamin results in confirmational change in both proteins (membrane bound EHD2 PDB 5MTV, Melo et al., 2017; membrane bound, constricted dynamin PDB 6DLU, Kong et al., 2018).

Structurally, EHD proteins share sequence similarity of up to 82% (Pohl et al., 2000) supporting the idea that EHDs could share a common function and lipid binding mechanism. EHD1-4 contain a stalk or helical region that is involved in membrane binding, a G-domain comprising the ATPase and oligomerization domain, and the specific EH domain, a Eps15 homologous protein sequence (Figure 3A, Daumke et al., 2007; Shah et al., 2014). EHD proteins are dimers which can be activated after membrane recruitment followed by ATP binding (Daumke et al., 2007; Hoernke et al., 2017; Melo et al., 2017). Mechanistically, the opening of the EHD dimer by repositioning of the EH domains results in the rearrangement of the stalk, freeing it to bind to the lipid bilayer. Detailed studies using EHD2 mutants in liposome binding assays accompanied with *in vivo* analysis showed that residues F322 and K327 are essential for correct membrane binding (Stoeber et al., 2012; Shah et al., 2014). ATP binding induces the oligomerization of EHD proteins resulting in liposome tubes that are decorated with EHD ring-like oligomers (Daumke et al., 2007; Melo et al., 2017). The diameter of these EHD tubes ranges between 20 and 80 nm indicating that EHD2 could form a ring surrounding the caveolar bud neck (Figure 3C). Ludwig et al. (2013) clearly localized EHD2 by immunogold labeling in EM section to the caveolar neck (Morén et al., 2012; Ludwig et al., 2013). In support of this, high resolution EM images show a distinct ring-like electron density at the caveolar neck (Popescu et al., 2006; Richter et al., 2008). A correlative imaging approach would be important to show that this density is indeed EHD2.

Upon ATP hydrolysis, the EHD oligomer disassembles and relocates from the caveolae membrane to the cytosol. Importantly, the ATP-dependent oligomerization step of EHD2 is key to stabilizing EHD2 at caveolae. Specifically, EHD2 mutants without the ATPase domain fail to oligomerize, and therefore fail to stabilize caveolae at the plasma membrane (Morén et al., 2012; Stoeber et al., 2012; Matthaeus et al., 2020). Furthermore, it was shown that in 3T3 fibroblasts EHD2 loss can be rescued by other EHD proteins (Yeow et al., 2017). However, a global EHD2 knockout mouse did not exhibit the same observations in adipocytes, fibroblasts, or endothelial cells (Matthaeus et al., 2019, 2020).

This raises the important question, why is EHD2, and in particular the ATP-dependent oligomerization of EHD2, essential for correct caveolae function and behavior? Several cellular studies have shown an increased caveolae mobility, endocytosis, and removal from the plasma membrane when the EHD2 gene is deleted or its ATP function impaired (Morén et al., 2012; Stoeber et al., 2012; Hoernke et al., 2017; Yeow et al., 2017). Endocytosis of transferrin receptor was not impaired in EHD2 knockout or EHD2 overexpressing cells (Pekar et al., 2012), highlighting the specific role of EHD2 in regulating caveolae dependent endocytosis. Additionally, overexpression of EHD2 mutants lacking their lipid binding or ATPase function also resulted in increased caveolae dynamics as a result of decreased caveolar membrane stabilization (Stoeber et al., 2012). In the last few years, two cell based studies showed an involvement of EHD2 in lipid accumulation (Li et al., 2016; Morén et al., 2019). Li et al. (2016) revealed an increased lipid droplet size in hepatocytes lacking EHD2. The authors proposed that EHD2 together with Rab10 and Ehbp1 is involved in lipolysis. Contrary, Moren et al. (2019) observed that EHD2 silencing in 3T3-L1 derived adipocytes reduced lipid droplet sizes. Additionally, Yeow et al. (2017) and Torrino et al. (2018) observed increased vulnerability to changes in membrane tension in EHD2 lacking fibroblasts or HeLa cells.

The generation of an EHD2 knockout mouse model helped to determine EHD2's caveolae function *in vivo*. We observed in mice that globally lacked EHD2 increased lipid droplet sizes in various tissue types although the total number of lipid droplets decreased (Matthaeus et al., 2020). Additionally, an increase in fatty acid uptake was detected in EHD2 knockout adipocytes and mouse embryonic fibroblasts. Detailed electron microscopy and tomography further supported the idea that EHD2 is essential for correct membrane stabilization of caveolae as EHD2-lacking tissues contained large numbers of detached caveolae (Matthaeus et al., 2019, 2020; Fan et al., 2020; Webb et al., 2020). Additionally, we observed an increased detachment of caveolae due to EHD2 removal resulting in reduced calcium entry and a resultant lack of activated eNOS and NO generation in endothelial cells. This lead to reduced blood vessel relaxation in EHD2 knockout mice and reduced running wheel endurance (Matthaeus et al., 2019). Taken together, these *in vivo* data clearly indicate that EHD2 oligomerization at the caveolar neck is an essential cell function with severe physiological consequences when EHD2 is missing or its ATPase function is impaired.

## GTP Dependent Dynamin Facilitated Membrane Scission of Caveolae

Caveolae detachment has been ascribed to dynamin catalyzed membrane scission. This raises the question of how dynamin drives fission of caveolae and how it is regulated. Dynamin is a well-known lipid binding GTPase that bends membranes and catalyzes fission. Structurally, it contains the N-terminal GTPase domain (G domain), followed by the central bundle signaling element (BSE), the stalk region, and the pleckstrin homology (PH) domain at the C-terminus (Figure 3B, Faelber et al., 2011). The stalk domain mediates dimerization, larger oligomers further include binding between BSE. Dynamin, with its 3 orthologs in mammalians, is expressed in all cell types from early embryonic time points on and is essential in clathrin mediated endocytosis (Ferguson and De Camilli, 2012). In particular, Dyn1 is found in the brain in high levels, Dyn3 in muscle, testis, brain and lung, and Dyn2 is a ubiquitous isoform expressed in many cell types. In clathrin mediated endocytosis, dynamin regulates the scission of clathrin coated membrane pits from the plasma membrane after coat assembly and elongation of the neck. However, dynamin and its related proteins such as interferon-inducible myxovirus resistance (Mx), Optic atrophy type 1 (Opa1) or Dynamin-1 like protein (Dnm1l) are involved in various other cellular processes within different organelles such as mitochondria fusion (Ferguson and De Camilli, 2012; Daumke and Praefcke, 2016).

How does dynamin facilitate membrane scission at caveolae? The scission of a phospholipid membrane requires the transition of the chemical energy gained during GTP hydrolysis into mechanical constriction to merge the lipid bilayers of the vesicle neck (Daumke and Praefcke, 2016). It is proposed that the hydrolysis of a few GTP molecules can provide the necessary energy for scission (Morlot et al., 2012). Structurally, the stalk region of dynamin forms ring-like oligomers surrounding the membrane (Kong et al., 2018). Following GTP binding, the GTPase domains dimerize, and GTP hydrolysis occurs resulting in a conformational change within the dynamin oligomer. This “power-stroke” is thought to pull the adjacent dynamin filaments along each other and thereby constricting the underlying membrane (Antonny et al., 2016; Daumke and Praefcke, 2016). Indeed, several *in vivo* studies showed the GTP dependent dynamin oligomerization and membrane scission at clathrin coated pits (Takei et al., 1995; Iversen et al., 2003; Grassart et al., 2014). Based on these results, it was concluded that caveolae detachment from the plasma membrane is also driven by dynamin. First, Schnitzer et al. (1996) showed a GTP dependent caveolae scission in a cell free assay indicating the involvement of a GTP handling enzyme (Schnitzer et al., 1996). Independently, Oh et al. (1998) and Henley et al. (1998) then described the GTP dependent caveolae internalization is mediated by dynamin (Henley et al., 1998; Oh et al., 1998). Furthermore, the involvement of dynamin was also observed in caveolae dependent albumin transcytosis (Shajahan et al., 2004). Importantly, overexpression of the non-GTP hydrolyzing dynamin mutant (Dyn2-K44A) abolished caveolae mobility, detachment, and trafficking from the plasma membrane in several cell types (Pelkmans et al., 2001; Senju et al., 2011; Oh et al., 2012). Taken together, these data indicated the involvement of dynamin in caveolae scission and detachment.

Some previous studies struggled to clearly localize dynamin to caveolar invaginations in different cell types. Using EM immunogold labeling, however, Dyn2 localization in caveolae was shown in kidney cells (Yao et al., 2005), and Dyn2 overexpressing MEFs or endothelial cells also showed co-localization with Cav1 (Shajahan et al., 2004; Matthaeus et al., 2020). The general difficulty of localizing dynamin could be caused by the fact that dynamin might only assembles at the caveolar neck shortly before internalization, followed by a fast re-location into the cytosol. To overcome this issue, the accumulation of non-hydrolyzing dynamin mutants at the caveolar neck was used to visualize dynamin with STORM microscopy (Platonova et al., 2015). Yao et al. (2005) could further identify a specific binding interaction between Cav1 and Dyn2. The exact role of dynamin at caveola is, however, still unclear.

Importantly, oligomerization of dynamin at the plasma membrane can only occur when thin membrane tubes are present. As previously described, EHD2 oligomerizes in rings around lipid bilayers in diameters ranging from 20 to 80 nm. Therefore, at the caveolar neck, we propose that EHD2 is key to create the necessary membrane structure for correct dynamin assembly and subsequent fission of caveolar invaginations (see model in Figure 3C). It was proposed previously, that EHD proteins are able to recruit dynamin to membrane tubes (Jakobsson et al., 2011), indicating an interaction between these two enzymes. However, based on the steric hindrance at the caveolar neck due to the large EHD2 oligomer, we suggest that before dynamin locates to the caveolae, EHD2 must start to disassemble (after ATP hydrolyzation) and relocate to the cytosol. Then, dynamin would be recruited to caveolae, followed by its oligomerization around the caveolar neck. The GTP dependent power stroke of dynamin would result in membrane scission. Later, detached caveolae would migrate to other intracellular organelles and dynamin would relocate to the cytosol. Further experiments, especially live cell high resolution imaging, are needed to test the temporal sequence of these events. Taken together, recent data shows the importance of GTP-dependent dynamin regulation during caveolae detachment. However, how these spatial and temporal mechanisms operate at the caveolar neck is still unknown and requires further study. Cleary, the interplay of EHD2 and dynamin at the membrane neck of caveolae is of particular interest for future work.

## Other Regulators for Caveolae Trafficking

### Rab GTPases in Caveolae Internalization and Trafficking

Besides dynamin and EHD2, several other GTP/ATP dependent proteins were assigned roles in caveolae trafficking. Specifically, Rab proteins have been linked with caveolae endocytosis. Rab proteins are small Ras-like GTPases that switch between the “on,” GTP bound—and the “off”—GDP bound—state and therefore temporally and spatially regulate the recruitment of various effector proteins such as vesicle coat proteins, membrane fusion complexes or motor proteins (see review Stenmark, 2009). The relatively high cytosolic GTP concentration (~0.5 mM; Traut, 1994) allows a very fast exchange of GDP with GTP securing the fast switching between the “off” and “on” state. It can be ventured that local changes in the GTP concentration might influence the activation of certain Rab molecules and their binding affinity to corresponding effector proteins. As Rab molecules are essential vesicle trafficking regulators it was expected to detect Rabs also at caveolar vesicles. Indeed, several studies focusing on caveolae endocytosis revealed that caveolae trafficking depends on Rab5, Rab7, and Rab11 (Pelkmans et al., 2004; Botos et al., 2008; Hayer et al., 2010b; Shvets et al., 2015). There, the Rab proteins direct the traffic of caveolae vesicles to early and late endosome and lysosome. However, to date, no caveolar specific Rab molecules were found which makes it challenging to study. Novel techniques analyzing protein-protein interaction such as SplitAPEX2 (Han et al., 2019) or fluorescence resonance energy transfer (FRET) could help to detect caveolae specific Rab molecules within distinct trafficking routes or organelles. Additionally, caveolae specific Rabs would help to determine the cellular implications for controlled caveolae trafficking at specific organelles.

### Regulation of Caveolae Internalization by Tyrosine Kinases

The characteristic lipid environment of caveolar invaginations leads to a specific set of membrane proteins including ATP handling enzymes localized to the caveolar membrane. One example is the distribution of Na/K-ATPase within different plasma membrane domains. A non-pumping Na/K-ATPase subpopulation is found in caveolae (Wang et al., 2004; Liang et al., 2007). Further, a study revealed the loss of Cav1 and caveolae at the plasma membrane after knockdown of Na/K-ATPase (Cai et al., 2008). Additional binding studies between purified Na/K-ATPase and Cav1 as well as cross-linking experiments demonstrated a direct interaction although within a low molar stoichiometry (Yosef et al., 2016; Nie et al., 2020). Knockdown of Na/K-ATPase also resulted in increased Src levels at the plasma membrane (Cai et al., 2008). It was shown previously that the tyrosine kinase Src is able to bind Cav1 leading to phosphorylation of the Cav1 residue tyrosine 14. After phosphorylation, Cav1 disassembles from the plasma membrane and caveolae internalization occurs (Parton et al., 1994; Lee et al., 2001). Several studies identified Cav1 as a substrate for Src which can be activated by various ligands and molecules, e.g., insulin or okadaic acid (Kiss and Botos, 2009), indicating that the phosphorylation of Cav1 plays an important role in caveolae internalization.

Besides Src, the tyrosine kinase Abl is also able to phosphorylate Cav1 tyrosine 14 in response to oxidative or tension stress (Sanguinetti and Corley Mastick, 2003). Interestingly, Abl is involved in the crosstalk between caveolae and stress fibers. Thereby, Abl together with the stress fiber regulator mDia1 (formin homology protein) and the F-BAR protein FBP17 regulates the formation and stabilization of caveolae at the plasma membrane (Echarri et al., 2012, 2019). Replica EM showing the cytosolic side of the plasma membrane (such as in Figure 1) illustrate that caveolae regularly locate in close proximity to actin filaments. Indeed, Filamin A was found to connect Cav1 with actin (Stahlhut and Van Deurs, 2000). Several studies showed that actin filaments are important for Cav1 and caveolae internalization, and that upon disruption of actin polymerization intracellular Cav1 trafficking is impaired (see detailed review, Echarri and Del Pozo, 2015). Additional, Pacsin2 and EHBP1 contain actin binding domains, and thereby both proteins are able to closely connect actin to caveolar membrane invaginations (Guilherme et al., 2004; Kostan et al., 2014). Of note, both proteins locate at the neck of caveolae suggesting that their actin binding motif might be an important regulator for caveolae mobility.

## Conclusion and Outlook

Caveolae internalization is found in many cells and tissues. Here, we summarized current concepts in caveolae trafficking and its role in physiology. As discussed above, essential steps during the internalization and migration are dependent on ATP or GTP (see also summary Figure 2). In the past, the small size of caveolae made it difficult to monitor their intracellular movements. Therefore, the application of recently developed high resolution live imaging methods will enable the full elucidation of intracellular caveolae trafficking throughout the cell. Novel imaging techniques such as super resolution light imaging and its combination with electron microscopy and tomography will allow for future detailed investigations of the caveolae life cycle and trafficking routes (Taraska, 2019). Also focus-ion beam scanning electron microscopy (FIB-SEM) could help to further dissect intracellular caveolae movement as this technique allows to visualize complete cell volumes in the highest resolution. A correlative approach to identify Caveolin and Cavin positive membranes would help to identify caveolar vesicles in the FIB-SEM stacks.

Of particular interest is the development or identification of caveolae specific cargos and receptors that are only internalized via caveolar endocytosis. This would allow caveolae-specific intracellular routes to be monitored and further identify novel factors involved in these pathways. New techniques for identifying the protein-protein interactions will provide additional insights in caveolae contact sites between various organelles. Finding these caveolae contact sites will help to determine how caveolae are involved in lipid metabolism and diseases. Another largely unexplored aspect in caveolae dynamics is the influence of local ATP/GTP concentrations. These changes can occur due to hypoxia. Indeed, cells growing under low oxygen levels show altered caveolae behavior. In adipocytes, it was observed that loss of oxygen results in decreased Cav1 expression and consequently reduced caveolae number (Regazzetti et al., 2015; Varela-Guruceaga et al., 2018). However, hypoxia in cancer cells and in the colon of the mouse intestine led to increased Cav1 expression (Wang et al., 2012; Xie et al., 2014; Bourseau-Guilmain et al., 2016). The oxygen sensitive transcription factor hypoxia-inducible factor (HIF1) was assigned to cause the changes in Cav1 gene expression (Wang et al., 2012; Xie et al., 2014; Bourseau-Guilmain et al., 2016; Varela-Guruceaga et al., 2018). This raises the possibility that caveolae could be sensitive to local ATP/GTP levels due to oxygen and nutrient deficiencies. The impact of low ATP levels on caveolae in cancer cells are of particular interest as caveolae are proposed to be involved in cancer progression. Taken together, energy requirements in caveolae trafficking are an important regulator for cellular metabolism and physiology. The extent of these mechanisms are not yet fully understood. Future work aimed at unraveling these questions will lead to a deeper understanding of the role these small plasma membrane organelles play in both health and disease.

## Author Contributions

CM wrote the manuscript and prepared figures. JT commented on and edited the manuscript.

## Conflict of Interest

The authors declare that the research was conducted in the absence of any commercial or financial relationships that could be construed as a potential conflict of interest.
